# Population health trends analysis and burden of disease profile observed in Sierra Leone from 1990 to 2017

**DOI:** 10.1186/s12889-022-14104-w

**Published:** 2022-09-22

**Authors:** Jolleen Zembe, Flavia Senkubuge, Tanita Botha, Tom Achoki

**Affiliations:** 1 Kofi Annan Global Health Leadership Fellowship, Africa CDC, African union commission, Addis Ababa, Ethiopia; 2 Department of Statistics, Faculty of Natural and Agricultural Sciences, University of Pretoria, Pretoria, South Africa; 3 Africa Institute for Health Policy, Nairobi, Kenya

**Keywords:** Non-communicable diseases (NCDs), Communicable, Maternal, Neonatal, And nutritional disease (CMNNs), Burden of disease, Sierra Leone

## Abstract

**Background:**

Sierra Leone, in West Africa, is one of the poorest developing countries in the world. Sierra Leone has experienced several recent challenges namely, a civil war from 1991 to 2002, a massive Ebola outbreak from 2014 to 2016, followed by floods and landslides in 2017.In this study, we quantified the burden of disease in Sierra Leone over a 27-year period, from 1990 to 2017.

**Methodology:**

In this descriptive study, we analysed secondary data from the Institute of Health Metrics and Evaluation, Global Burden of Disease (GBD) study. We quantified patterns of burden of disease, injuries, and risk factors in Sierra Leone. We report GBD data and metrics including mortality rates, years of life lost and risk factors for all ages and both sexes from 1990 to 2017.

**Results:**

From 1990 to 2017, trends of mortality rates for all ages and sexes have declined in Sierra Leone although mortality rates remain some of the highest when compared to other developing countries. The burden of communicable, maternal, neonatal, and nutritional (CMNN) diseases are greater than the burden of non-communicable diseases (NCDs) due to the prevalence of endemic diseases in Sierra Leone. The most important CMNNs associated with premature mortality included respiratory infections, neglected tropical diseases, malaria, and HIV-Aids. Life expectancy has increased from 37 to 52 years.

**Conclusion:**

Sierra Leone’s health status is gradually improving following the civil war and Ebola outbreak. Sierra Leone has a double burden of disease with CMNNs leading and NCDs progressively increasing. Despite these challenges, Sierra Leone has promising initiatives and programs pursuing the Universal Health Coverage 2030 Sustainable Developmental Goals Agenda. There is need for accountability of available resources, clear rules and expected roles for non-governmental organisations to ensure a level playing field for all actors to rebuild the health system.

**Supplementary Information:**

The online version contains supplementary material available at 10.1186/s12889-022-14104-w.

## Background

The world is experiencing rapid health, demographic, and epidemiologic transitions. Many developing countries are monitoring their health trends due to poor health outcomes from infectious diseases and an increase in chronic diseases. Sierra Leone is a developing country with a history grounded in the slave trade in the eighteenth century to a civil war, which lasted for ten years. Despite prolonged periods of conflict, Sierra Leone has made great strides towards achieving political stability from a history of long periods of conflict. Sierra Leone has an ailing economy which is slowly growing following the civil war which ended in 2002. Sierra Leone’s gross domestic product is growing between 4 and 7% annually [1]. As a consequence of political instability, Sierra Leone has a dysfunctional health system which remains a challenge [2]. Sierra Leone has a population of about 7.4 million with a reported of growth rate of 2.18% in 2017 [3].

The Institute for Health Metrics and Evaluation (IHME) has been measuring ongoing Global Burden of Disease (GBD) for 27 years. The GBD study measures the most important health problems in each country and how health systems are responding to their health problems [3]. The GBD quantified quantifies mortality caused by major health problems, injuries, risk factors by age and sex [3, 4]. From 1990 to 2017, the incidence and prevalence of 354 causes in 195 countries were thoroughly analyzed [3, 4]. The GBD study provides evidence and motivation for governments to allocate resources and set relevant health agendas [3, 5].

Sierra Leone made remarkable strides and laudable progress towards the implementation of the Millennium Developmental Goals from around 2002 despite the civil war and an Ebola virus outbreak in May 2014 [6]. Sierra Leone’s government attempted to address the health needs of their population by increasing the healthcare finance budget by 34%, sourcing 86.5% of necessary funds from external partners for the Free Health Initiative [7]. The Free Health Initiative 2010 for women and children increased and improved health access and coverage to address high morbidity and mortality in women and children [8].

In this study, we report on the GBD study focusing on Sierra Leone from 1990 to 2017 (27 years). In Sierra Leone, the burden of disease is characterised by a combination of persistent, emerging and re-emerging infectious diseases and increasing chronic conditions and injuries. Sierra Leone is experiencing a double burden of non-communicable diseases (NCDs) and communicable diseases, maternal, neonatal and nutritional disease (CMNNs). As in most developing countries, the burden of CMNNs seems to be decreasing but with fluctuations caused by persistent malaria. The burden of NCDs also seems to be steadily decreasing but hypertension, alcohol and substance abuse are prevalent in the society and NCDs are predicted to increase. We analysed the GBD data metrics and disease trends to describe changing burdens of CMNNs and NCDs over a 27-year period in Sierra Leone.

## Methods

We conducted a descriptive study using secondary data from IHME GBD database [3]. Data from 1990 to 2017 were extracted on the causes of mortality and morbidity for all age groups and both genders. The GBD estimates burden of disease using quality-controlled, bias-corrected data sources, including country wide surveys, birth and death registration systems, census and disease surveillance which are released annually. The data are analysed using standardised statistical estimation and cross-validated to assess model performance [3]. Sampling and non-sampling error in the data and model assumptions are accounted for by reporting 95% uncertainty intervals (UIs) for all GBD estimates. The UIs are derived from the 2.5th and 97.5 percentiles of 1 000 draws [3]. Complete information on the GBD data sources are available from the Global Health Data Exchange. Data can be explored and visualised on the IHME website. The GBD framework also classifies causes of health loss into mutually exclusive and collectively exhaustive categories organised in a four-level hierarchy [3]. The causes of health loss are first organised into three primary categories namely CMNNs, NCDs and injuries. These broad categories are divided further into increasingly more detailed categories in a consistent and comprehensive manner [3]. Standard estimates for different causes of health loss are produced for different sexes and age groups by country, enabling useful comparisons.

### Mortality estimates

The IHME GBD estimates mortality rates of adults of all ages and both sexes including children under five [9]. Data for children under five and adults are separated using Gaussian and spatiotemporal regressions. Cause-specific mortality is estimated using standard data sources which show cause of death including death registrations, reports from autopsies and surveillance [9]. Data with no cause of death are allocated garbage codes and redistributed using standard algorithms. The Cause of Death Ensemble mode on the IHME website uses country-level covariates and builds models which are combined and evaluated to provide the most robust estimates for cause-specific mortality. Models for cause-specific mortality are combined and corrected to be internally consistent with estimates of all-cause mortality using the cause of death correction process, Cod Correct [3]. In this study we will focus on YLLs and contributing risk factors.

### Years of life lost

The Years of life lost summarizes years lost to premature death, at which age death occurred and the frequency of deaths [9]. YLL is expressed per 100,000 population [3]. YLL is calculated using the formula: $YLL={{N}_{\left(cause\,of\,death+age+year\right)}}\times {{L}_{\left(sex+age\right)}}$, where N = mortality and L = standard life expectancy at the age at which the death occurred [3]. The formula was developed through consultations, collaboration and research with experts and is supported by the World Global Health Estimates which are curated by the World Health Organisation [9, 10]. In 2010, the GBD study simplified the calculation. The values were acknowledged and adopted by the World Health Organisation (WHO) [10].

## Results

**Fig. 1:**
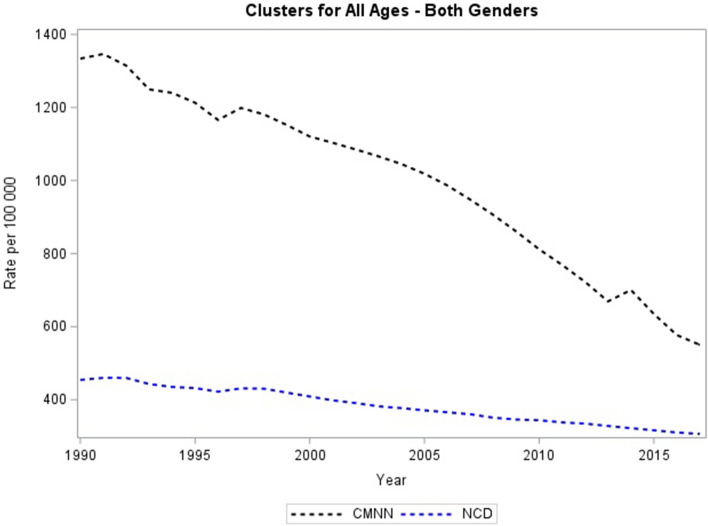
Trends in death rates of CMNNs and NCDs all ages, sexes for Sierra Leone from 1990–2017. Mortality rates for the population of Sierra Leone

These results report the death rates, per 100 000, for CMNN and NCD (Fig. 1). Between 1990 and 2017, the burden of both CMNNs and NCDs declined for men and women this is depicted by the overall decrease in death rates. By 2017, Sierra Leone still had a larger burden of CMNNs than NCDs, although the burden of CMNNs had declined markedly since 1990 (Fig. 1). In 1990, men had a greater burden of NCDs, but by 2017 the gap between men and women had narrowed. The burden of CMNNs dropped remarkably for both men and women (Fig. 1). Despite the declining burden of CMNNs, men were still more affected than women. We noted increases in CMNNs in 1997 and 2014, for both men and women, hinting at events that destabilised the health system.

### Top ten Trends in CMNNs and NCDs (YLLs)

**Fig. 2:**
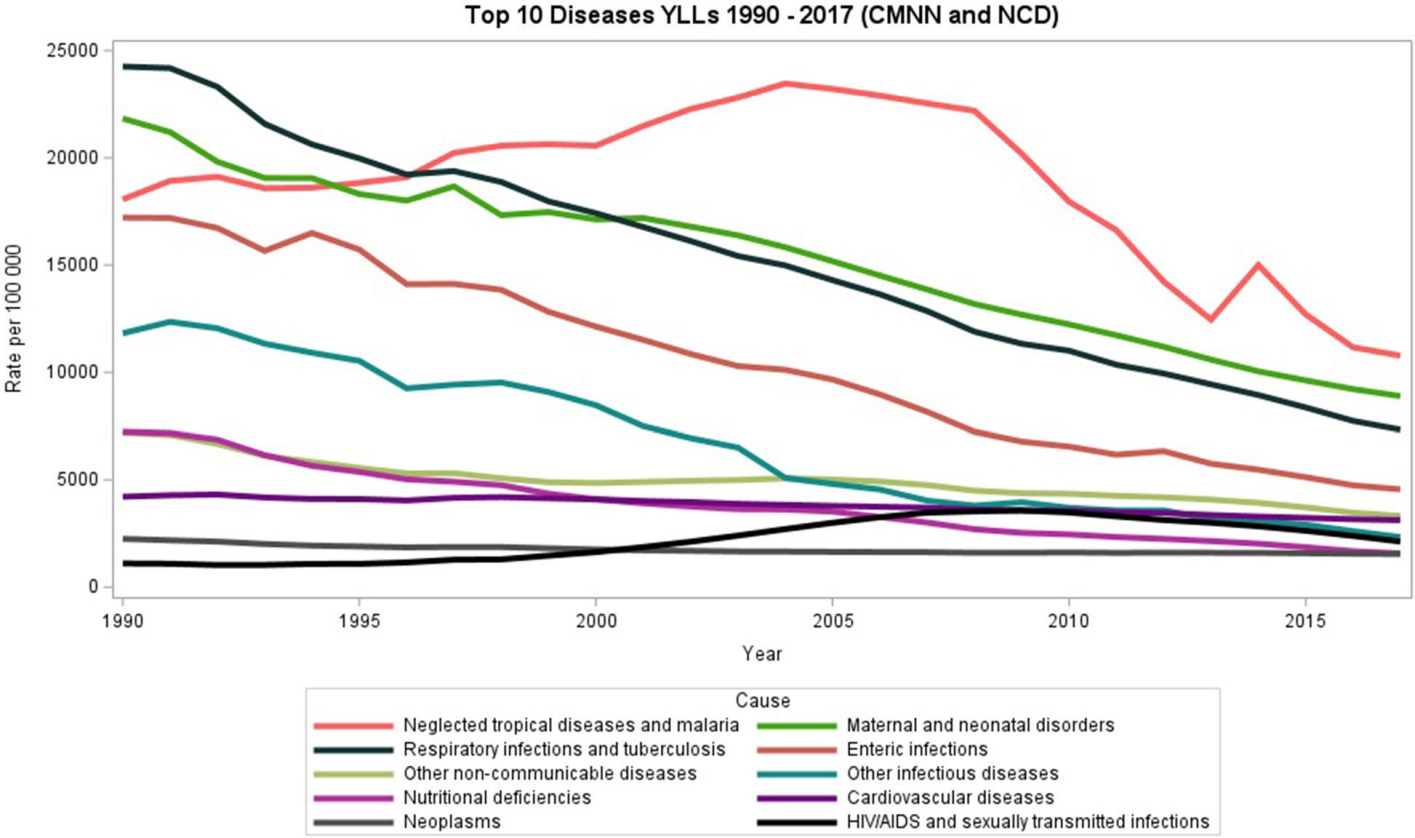
Trends (YLLs) for top CMNNs and NCDs in Sierra Leone from 1990—2017

The following results reports on the top 10 diseases for CMNN and NCD combined, for all ages and both genders, reported in YLL rate per 100 000. The most important contributor to YLLs in Sierra Leone over the study period were neglected tropical diseases including malaria (Fig. 2). In 1990, these diseases caused about YLLs 18,000 /100000 population with 95% (UI) 7,619.00 lower limit to 35,144.00 upper limit.There was a steady increase to YLLs 20,000 /100000 population in 2000 and peaked at YLLs 24,000 per 100,000 in 2004 with 95% (UI) ranging from (LL) 9,840.41- 35,777.66 (UL). This peak lasted until 2008, when YLL due to neglected tropical diseases and malaria started to decline however they have still remained high in the period under review (Fig. 2).

Maternal and neonatal deaths were the 2^nd^ largest contributor to YLLs in the period under review. Although maternal and neonatal deaths have declined steadily from 1995 to 2017, these deaths have remained an important contributor to total YLLs. In 2000, maternal and neonatal deaths (YLLs 17,000/100000), 95%(UI) ranging from (LL)14,308-(UL)19,601 overtook respiratory infections and tuberculosis (YLLs16000/100000) with 95% (UL) ranging from (LL)14,308 -19.601 (Fig. 2).

The burden of respiratory infections peaked in 1990 accounting for an estimated YLLs 25,000 per 100,000 population (Fig. 2) with 95% (UI) ranging between (LL)16,086.00-(UL)27,27,301.00. The burden of respiratory infections declined steadily to about 8000 YLLs deaths per 100,000 population in 2017. In 2017, respiratory infections remained the third largest contributor to YLLs,95% uncertainty intervals ranging from (LL) 5,513 –(UL) 10,449. The burden of YLL due to enteric diseases and other infectious diseases have declined dramatically over the 27-year review period. Yearly lives lost due to HIV/AIDS and sexually transmitted infections gradually increased from 1990—2017.

### Risk factors of CMNNs

Table 1 summarizes the risk factors for CMNNs. Throughout the study period, child and maternal malnutrition-related problems were ranked first. Secondly, contaminated drinking water, inadequate sanitation and a lack of handwashing facilities continue to be an issue in Sierra Leone. Exposure to air pollution was rated as the third most important risk factor for YLLs. Between 2000 and 2017, the importance of cigarette consumption declined from fourth to sixth place. Over the 27-year study period, the importance of risky sexual practices grew from seventh to fourth. Fasting glucose levels were first placed sixth, but then dropped to eighth position. Intimate partner violence increased in prominence, increasing from eighth to seventh place (1990–2000). From 1990 to 2017, the importance of drug use remained constant, ranking ninth.

**Table 1:**
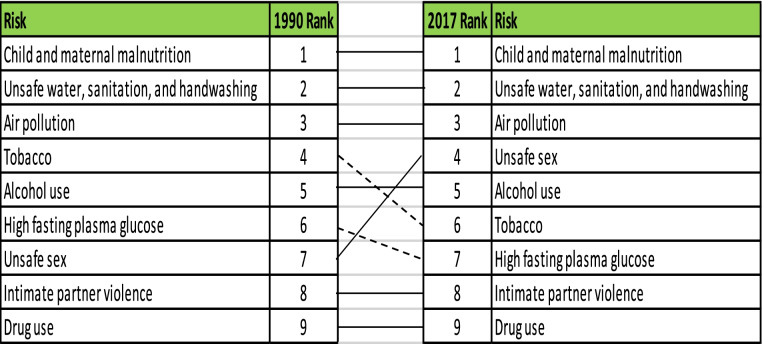
Risk factors contributing to CMNNs in Sierra Leone from 1990 to 2017

### Risk Factors of NCDs

Table 2 displays the risk factors which predispose the population of Sierra Leone to NCDs from 1990 and 2017. The GBD study identifies 16 risk factors for YLLs, noncommunicable diseases in Sierra Leone [3]. During 1990 and 2017, the most important risk factors for NCDs were high systolic blood pressure and dietary hazards (Table 2). In 2000, fasting glucose became the third most important risk factor, a position it held for seventeen years. Tobacco and alcohol usage have diminished in relevance as risk factors. Tobacco use was ranked third in 1990, fifth in 2000, and finally sixth in 2010. In 2010, alcohol consumption slipped from seventh to ninth place, where it remained until 2017. From 1990 to 2017, all drug consumption was rated tenth. From ninth place in 1990 to fourth place in 2017, a high body mass index increased in prominence as a risk factor. From 1990 to 2017, environmental risks and child and maternal malnutrition maintained consistent rankings (Table 2).

**Table 2:**
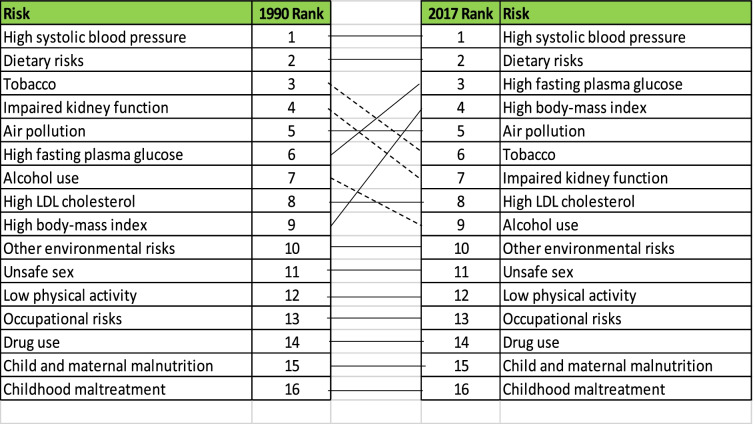
Risk factors contributing to non-Communicable disease for 1990 -2017

## Discussion

In this GBD study, we describe the burden of disease in Sierra Leone from 1990 to 2017. Overall, the burden of disease improved significantly resulting in decreased mortality rates. According to the trend analysis Sierra Leone is faced with a dual burden of disease, with CMNNs contributing about 65% while NCDs account for about 29% and 6% represent injuries.,the CNMMs and NCDs [11, 12]. In Sierra Leone, CMNNs continue to be a problem due to the prevalence of endemic diseases [3]. The most important CMNNs are respiratory infections, neglected tropical diseases and malaria, and maternal and neonatal disease, they contribute significantly to (YLLs), mortality and disablity [3]. The burden of NCDs was low compared to CMNNs, a trend which is likely to change as the health system recovers and populations age. The end of the Sierra Leone civil war brought the government, international partners, stakeholders, and civil society together to start reconstructing the health system. The recovering health system and implementation of health policy interventions has resulted in the decline of YLLs due to CMNNs and NCDs. Our study shows that Sierra Leone has made progress in population health outcomes despite multiple challenges.

The Sierra Leone civil war took place from 1991 to 2002, lasted 11 years and left more than fifty thousand people dead. The civil war would have contributed to burden of disease in many ways, including an increase in injuries. Mortality rates due to NCDs peaked between 1990 and 1994, reflecting the potential impact of the civil war. Mortality rates have declined consistently after the end of the civil war, suggesting efforts to rebuild the health system. Contrary to this the two- year Ebola outbreak contributed to a slight increase in mortality in 2014 [3].

Sierra Leone is dominated by communicable,maternal neonatal diseases since 1990 to date [3]. The burden of CMNNs is high when compared to other countries [3]. The burden of CMNNs peaked in 1990 and 1992 and can be attributed to persistent endemic malaria [13]. Malaria is the leading cause of death and poses a serious threat to the whole population [14, 15]. Sierra Leone health services treat approximately 2,240,000 outpatients annually for malaria and almost half of these are children under the age of five years [14, 16]. Malaria mortality was estimated at approximately 4.4% of pregnant women and 17% of children. Malaria contributes to 40% of hospitalised morbidity in all ages and 37% of children under five [14, 15]. Malaria has been a priority and remains on Sierra Leones health agenda since 1990 but the civil war in 1991 the civil war resulted in the displacement and uncoordinated efforts of malaria control [14]. In 2004, Sierra Leone launched their first National Malaria Strategic Plan (2004–2008), which was funded by the Global fund, nevertheless they continue to fight malaria [14]. The National Malaria Control programme within the Ministry of Health continues to distribute insecticide-treated nets and provide access to malaria preventive therapy. Key challenges include a lack of human resources to coordinate and implement the programme in the rural districts and a limited supply chain at all levels [14, 15].

Sierra Leone was reported to have the highest Maternal mortality ratios in the world at 1360 deaths per 100,000 live births in 2015, which far exceeds the MDG targets of 450 deaths per 100,000 births [12, 17]. In Sierra Leone, children under five years suffer high mortality rates with 120 deaths per 1000 children [15]. To achieve Millennium Developmental Goal 5a, the government made commendable efforts to reduce maternal mortality by 75 percent, but these efforts were hampered by the effects of the civil war and the Ebola outbreak, which crippled the infrastructure and economy [6]. In 2010, the Sierra Leone government launched the Free Health Care Initiative to reduce mortality and morbidity due maternal and neonatal disorders [8, 12]. The Free Health Care Initiative has contributed to a significant improvement in the health system access and coverage as shown by the statistics in the study [8]. Similarly successful health care Initiatives and policies were implemented in Burundi and Ghana [8].

In Sierra Leone, respiratory diseases and tuberculosis, HIV/AIDS, and enteric infections are the major drivers of YLLs [3]. In the 2016 WHO Global TB Report, Sierra Leone was ranked ninth in the world in terms of incidence per capita [15]. In 1990, the German Leprosy Relief Association assisted the Ministry of Health and Sanitation to establish the National Leprosy and Tuberculosis Control Programme to monitor the surveillance of tuberculosis control activities [13, 18]. Sierra Leone continues to have one of the highest tuberculosis burdens in the world despite the fact that treatment is free and readily available [15, 19]. Sierra Leone opened its first drug-resistant tuberculosis treatment centre at Lakka Government Hospital in 2017 [19]. Shortage of human resources and long distances from health facilities are the main challenge in this program [13]. Nonetheless, new recommendations, constant monitoring and surveillance of the National Tuberculosis Program remain necessary [19].

Enteric diseases are most prevalent in children under the age of five and account for around 12% of all child deaths in Sierra Leone [20]. Sierra Leone added the rotavirus vaccine to its immunization schedule to combat diarrhoeal infections on March 28, 2014, in an effort to address this issue [20]. The government continues to prioritise prevention and treatment of childhood illnesses.

The burden of HIV/AIDS and sexually transmitted infections (STIs) increased over the 27 years [3]. The prevalence of HIV/AIDS is approximately 1.7%.The prevalence of HIV/AIDS prevalence in Freetown, the capital city [21]. It affects age group ranging from 15–49 years all sexes [21]. In 2013 and 2014, commercial sex workers were responsible for 40% of newly infected HIV patients [22]. The Sierra Leone government is stepping up efforts to test, prevent, treat and increase awareness with the support of the WHO, Global Fund and many other partners. The Sierra Leone government has implemented a national HIV AIDS strategic plan 2016–2020, including programmes such as Prevention of Mother to Child Transmission [19].

As a developing country with a young population, the risk factors associated with YLLs due to CMNNs are linked to the health and wellbeing of younger age groups. The most important risk factors for CMMN YLLs were environmental risk factors including child and maternal nutrition, unsafe water and sanitation and exposure to air pollution. Less important risk factors included lifestyle risk factors such as alcohol and tobacco use, drug use and intimate partner violence. In Sierra Leone, environmental risk factors are being addressed on various fronts. These lifestyle risk variables were associated with a relatively small number of deaths; for example, cigarette smoking was associated with 5% of YLLs [3]. Although the number of YLLs connected with these risk factors is still small, it is increasing and requires monitoring by local organizations.

The global prevalence of NCDs is expected to grow by 25% globally by 2030 [23]. In 2008, the WHO estimated that NCDs accounted for 18% of fatalities in Sierra Leone, followed by cardiovascular disease at 7%, cancer at 3%, diabetes at 1%, and chronic respiratory illness at 2% [24]. Sierra Leone was also predicted to experience an increase in Non-Communicable diseases [23–25]. In 2012, mortality from NCDs increased to 26%. Sierra Leone's government developed its first strategic plan and policy for NCDs in 2013, in response to the World Health Organization's global status report on NCDs. The 2013–2017 strategic plan, of Sierra Leone aimed to mitigate the burden of NCDs such as cardiovascular disease, chronic lung disease, diabetes mellitus, obesity, cancer, sickle cell disease, mental disorders, and epilepsy [24, 26]. By 2014, the incidence of NCDs had reduced across all age groups and sexes which shows the strategy had positive results [25].

The burden of NCDs remained constant between 2005 and 2017. In our study, most YLLs due to NCDs can be attributed to cardiovascular related diseases and neoplasms contributing to approximately 9% of NCD deaths [24]. There is evidence that NCDs are increasing. In 1993, 68% of hospitalisations at Freetown hospital were admitted due stroke [24]. In 1994, 25% of the population above 50 years of age were estimated to be hypertensive [24]. A review of death certificates issued between 1983 and 1992, showed an increase in deaths related to hypertension in Sierra Leone [24]. There is little information on the prevalence of cancer in Sierra Leone, even though our study reported that neoplasms were among the top ten causes of mortality [24]. In Sierra Leone, recording and reporting of data on NCDs remains inconsistent even though there is a ministerial department responsible for NCDs [24].

Sierra Leone suffered an Ebola outbreak in 2014 and 2015, led to inadequate quality surveillance data on the incidences, cases and deaths of NCDs [27]. The Ebola outbreak occurred when the government was transitioning from hospital care for NCDs to management, treatment and care in primary health care facilities [27]. Following the Ebola outbreak, significant reporting systems focusing on morbidity and risk factors for NCDs were put in place. Although policies are being developed by the government, there seems to be little funding for treating and controlling NCDs [27].

Dietary risks are also associated with YLLs due to NCDs in Sierra Leone. A nutritional survey done in 2014 revealed that more than 25% of children younger than five years old had stunted growth [24, 27]. Glucose has recently become an important risk factor NCD associated YLLs and is growing in importance. High fasting plasma glucose is an indicator of diabetes mellitus. The prevalence of diabetes in Sierra Leone has also increased from 2.4% in 1997 to 7% in 2014 [24].

Tobacco use is an important risk factor of NCDs, including cardiovascular disease, respiratory diseases and lung cancers [24]. In Sierra Leone, 14.3% of men and 1.4% of women, comprising 34% of people, smoke more than 10 cigarettes a day [24]. Sierra Leone signed the WHO Framework Convention on Tobacco Control in May 2009, with the objective of reducing tobacco consumption, and the Ministry of Health and Sanitation adopted a National Tobacco Control Strategic Plan in 2012.In addition to problems of hypertension, glucose and substance abuse is the fact that Sierra Leoneans engage in low to moderate physical activity. The importance of high body mass index as a risk factor jumped from 9th in 1990 to 5th place in 2017 [3]. The burden of NCDs remains low compared to CMNNs, which may contribute to few resources being allocated to preventing NCDs at this point.

### Limitations

There is a general dearth of information due to the multiple systems utilised by the Ministry of Health and the private sector, Sierra Leone's health information systems are still fragmented and multi-operating, causing it to lag behind [13]. The district’s health information system and integrated disease surveillance and response systems are not well-coordinated, so the data's veracity is generally sceptical. The information on non-communicable disease is limited [13]. To strengthen research, it is necessary to strengthen information monitoring and evaluation tools.

Access to high-quality, efficient service delivery remains a challenge due to lack of financial resources, essential medicines, and equipment. Sierra Leone continues to struggle with human resource shortages and misdistribution in rural and urban areas [28]. The country is also experiencing massive migration of highly specialised health workers. An estimated 300 health workers died during the Ebola outbreak [28].

## Conclusion

We described the burden of disease profile in Sierra Leone. We described the trends and patterns of CMNNs and NCDs in Sierra Leone for the period 1990 to 2017. The burden of disease, expressed as YLLs, in Sierra Leone declined from 1990 to 2017. During this time, the most dramatic decreases were seen in YLLs attributed to CMNNs. YLLs due to CMNNS remain higher than NCDs due the presence of endemic diseases including respiratory infections, neglected tropical diseases and malaria. Maternal and neonatal disease also contributed to YLLs. The high burden of these conditions is driven by environmental risk factors including inadequate nutrition, unsafe water and sanitation and air pollution. The burden of NCDs was represented by cardiovascular disease and to a lesser extent neoplasms. Although low compared to CMNNs, the Sierra Leonean government should monitor the impact of NCDs, to inform health promotion strategies. As the health system recovers from the civil war and the Ebola outbreak, the quality of health care will improve, and the population will age. As with other developing countries, aging populations are associated with a greater burden of NCDs. The end of the civil war brought together the government, international partners, stakeholders, and civil society to start reconstructing the health system and implementing health policy interventions which have contributed to the decline of CMNNs and NCDs.

## Supplementary Information

**Additional file 1: Supplementary Figure 1.** CMNN and NCD combined mortality rates. **Supplementary Figure 2.** Top 10 Diseases for CMNN and NCD combined. **Supplementary Table 1.** CMNNs risk factors. **Supplementary Table 2.** NCD Risk factors.

## Data Availability

All data generated or analysed during this study are included in this published article and its supplementary information files.

## Abbreviations

AIDS: Acquired Immune Deficiency Syndrome
CMNN: Communicable Maternal Neonatal Nutritional Diseases
DALY: Disability-Adjusted Life Years
DHIS: District Health Information System
GBD: Global Burden of Disease
HC: Health Centre
HIV: Human Immunodeficiency Virus
HRH: Human Resource for Health
IHME: Institute for Health Metrics and Evaluation
MoH: Ministry of Health
MDG: Millennium Development Goal
NCD: Non-Communicable Disease
NGO: Non-Governmental Organization
SDG: Sustainable Development Goal
TB: Tuberculosis
UN: United Nations
UI: Uncertainty interval
UL: Upper limit
LL: Lower limit
WHO: World Health Organization
YLL: Years of Life Lost
YLD: Years of Life Lost to Disability
