# Correction to: Population health trends analysis and burden of disease profile observed in Sierra Leone from 1990 to 2017

**DOI:** 10.1186/s12889-022-14366-4

**Published:** 2022-10-31

**Authors:** Jolleen Zembe, Flavia Senkubuge, Tanita Cronje, Tom Achoki

**Affiliations:** 1 School of Health Systems and Public Health, Faculty of Health Sciences, University of Pretoria, Pretoria, South Africa; 2 Kof Annan Global Health Leadership Fellowship, Addis Ababa, Ethiopia; 3 Department of Statistics, Faculty of Natural and Agricultural Sciences, University of Pretoria, Pretoria, South Africa; 4 Africa Institute for Health Policy, Nairobi, Kenya

**Correction to**: ***BMC Public Health*22, 1801 (2022). **10.1186/s12889-022-14104-w.

The original publication of this article contained incorrect affiliations; the correct affiliations are available in this correction article. The original article has been updated.

