# Supplementary material for: Population health trends analysis and burden of disease profile observed in Sierra Leone from 1990 to 2017
Source: BMC Public Health. 2022 Sep 22;22:1801. doi: 10.1186/s12889-022-14104-w (PMC9494828; doi:10.1186/s12889-022-14104-w)
Supplement: Supplementary file 1 — Additional file 1: Supplementary Figure 1. CMNN and NCD combined mortality rates. Supplementary Figure 2. Top 10 Diseases for CMNN and NCD combined. Supplementary Table 1. CMNNs risk factors. Supplementary Table 2. NCD Risk factors. [file 12889_2022_14104_MOESM1_ESM.docx]

**APPENDIX**

**Supplementary Figure 1 : CMNN and NCD combined mortality rates**

These results report the death rates, per 100 000, for CMNN and NCD. The results include all ages but are split by gender when reported


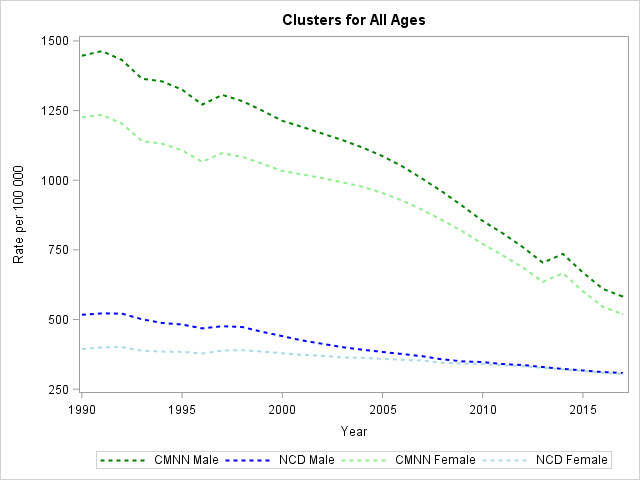


**Supplementary Figure** **2: Top 10 Diseases for CMNN and NCD combined**


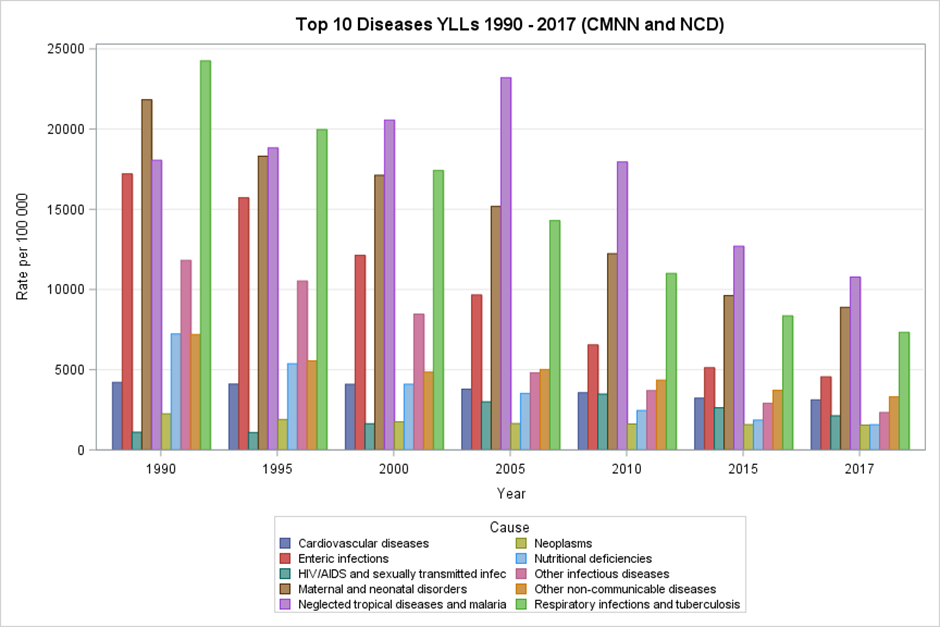


Figure 2: The following results reports on the top 10 diseases for CMNN and NCD combined, for all ages and both genders, reported in YLL rate per 100 000. The top 10 was determined by considering the average rank of each disease across all years and selecting the top 10 ranked.

**Risk factors for NCD and CMNN Combined**

The following results report the risk factors, for all ages both genders, measured in YLL rate per 100 000 for all NCD and CMNNN causes. The data downloads allow for a split in the two or a combination or NCD, CMNN and Injuries

**Supplementary Table 1: CMNNs risk factors**

|  | **Rank** | | | | | | | |  |
| --- | --- | --- | --- | --- | --- | --- | --- | --- | --- |
| **Risk** | **1990** | **1995** | **2000** | **2005** | **2010** | **2015** | **2016** | **2017** | |
| High systolic blood pressure | 1 | 1 | 1 | 1 | 1 | 1 | 1 | 1 | |
| Dietary risks | 2 | 2 | 2 | 2 | 2 | 2 | 2 | 2 | |
| High fasting plasma glucose | 6 | 6 | 3 | 3 | 3 | 3 | 3 | 3 | |
| Air pollution | 5 | 4 | 4 | 4 | 4 | 4 | 4 | 5 | |
| Tobacco | 3 | 3 | 5 | 5 | 5 | 6 | 6 | 6 | |
| Impaired kidney function | 4 | 5 | 6 | 6 | 6 | 7 | 7 | 7 | |
| Alcohol use | 7 | 7 | 7 | 7 | 7 | 9 | 9 | 9 | |
| High body-mass index | 9 | 9 | 9 | 9 | 8 | 5 | 5 | 4 | |
| High LDL cholesterol | 8 | 8 | 8 | 8 | 9 | 8 | 8 | 8 | |
| Other environmental risks | 10 | 10 | 10 | 10 | 10 | 10 | 10 | 10 | |
| Unsafe sex | 11 | 11 | 11 | 11 | 11 | 11 | 11 | 11 | |
| Low physical activity | 12 | 12 | 12 | 12 | 12 | 12 | 12 | 12 | |
| Occupational risks | 13 | 13 | 13 | 13 | 13 | 13 | 13 | 13 | |
| Drug use | 14 | 14 | 14 | 14 | 14 | 14 | 14 | 14 | |
| Child and maternal malnutrition | 15 | 15 | 15 | 15 | 15 | 15 | 15 | 15 | |
| Childhood maltreatment | 16 | 16 | 16 | 16 | 16 | 16 | 16 | 16 | |
|  |  |  |  |  |  |  |  |  | |

**Supplementary Table 2: NCD Risk factors**

|  | **Rank** | | | | | | | |
| --- | --- | --- | --- | --- | --- | --- | --- | --- |
| **Risk** | **1990** | **1995** | **2000** | **2005** | **2010** | **2015** | **2016** | **2017** |
| Child and maternal malnutrition | 1 | 1 | 1 | 1 | 1 | 1 | 1 | 1 |
| Unsafe water, sanitation, and handwashing | 2 | 2 | 2 | 2 | 2 | 2 | 2 | 2 |
| Air pollution | 3 | 3 | 3 | 3 | 3 | 3 | 3 | 3 |
| Alcohol use | 5 | 5 | 5 | 5 | 5 | 5 | 5 | 5 |
| Tobacco | 4 | 4 | 4 | 6 | 6 | 6 | 6 | 6 |
| Unsafe sex | 7 | 7 | 6 | 4 | 4 | 4 | 4 | 4 |
| High fasting plasma glucose | 6 | 6 | 7 | 8 | 8 | 8 | 8 | 7 |
| Intimate partner violence | 8 | 8 | 8 | 7 | 7 | 7 | 7 | 8 |
| Drug use | 9 | 9 | 9 | 9 | 9 | 9 | 9 | 9 |
